# Sequence composition of BAC clones and SSR markers mapped to Upland cotton chromosomes 11 and 21 targeting resistance to soil-borne pathogens

**DOI:** 10.3389/fpls.2015.00791

**Published:** 2015-10-02

**Authors:** Congli Wang, Mauricio Ulloa, Xinyi Shi, Xiaohui Yuan, Christopher Saski, John Z. Yu, Philip A. Roberts

**Affiliations:** ^1^Department of Nematology, University of California, RiversideRiverside, CA, USA; ^2^Key Laboratory of Mollisols Agroecology, Northeast Institute of Geography and Agroecology, Chinese Academy of SciencesHarbin, China; ^3^Plant Stress and Germplasm Development Research Unit, USA - Agricultural Research ServiceLubbock, TX, USA; ^4^Key Laboratory of Soybean Molecular Design Breeding, Northeast Institute of Geography and Agroecology, Chinese Academy of SciencesHarbin, China; ^5^Clemson University, Genomics InstituteClemson, SC, USA; ^6^USA - Agricultural Research Service, Southern Plains Agricultural Research Center, College StationTX, USA

**Keywords:** *Gossypium hirsutum*, genetic and physical mapping, resistance-rich cluster, resistance stress element, root-knot nematode, Fusarium wilt, soil-borne disease

## Abstract

Genetic and physical framework mapping in cotton (*Gossypium* spp.) were used to discover putative gene sequences involved in resistance to common soil-borne pathogens. Chromosome (Chr) 11 and its homoeologous Chr 21 of Upland cotton (*G. hirsutum*) are foci for discovery of resistance (R) or pathogen-induced R (PR) genes underlying QTLs involved in response to root-knot nematode (*Meloidogyne incognita*), reniform nematode (*Rotylenchulus reniformis*), Fusarium wilt (*Fusarium oxysporum* f.sp. *vasinfectum*), Verticillium wilt (*Verticillium dahliae*), and black root rot (*Thielaviopsis basicola*). Simple sequence repeat (SSR) markers and bacterial artificial chromosome (BAC) clones from a BAC library developed from the Upland cotton Acala Maxxa were mapped on Chr 11 and Chr 21. DNA sequence through Gene Ontology (GO) of 99 of 256 Chr 11 and 109 of 239 Chr 21 previously mapped SSRs revealed response elements to internal and external stimulus, stress, signaling process, and cell death. The reconciliation between genetic and physical mapping of gene annotations from new DNA sequences of 20 BAC clones revealed 467 (Chr 11) and 285 (Chr 21) *G. hirsutum* putative coding sequences, plus 146 (Chr 11) and 98 (Chr 21) predicted genes. GO functional profiling of Unigenes uncovered genes involved in different metabolic functions and stress response elements (SRE). Our results revealed that Chrs 11 and 21 harbor resistance gene rich genomic regions. Sequence comparisons with the ancestral diploid D_5_ (*G. raimondii*), A_2_ (*G. arboreum*) and domesticated tetraploid TM-1 AD_1_ (*G. hirsutum*) genomes revealed abundance of transposable elements and confirmed the richness of resistance gene motifs in these chromosomes. The sequence information of SSR markers and BAC clones and the genetic mapping of BAC clones provide enhanced genetic and physical frameworks of resistance gene-rich regions of the cotton genome, thereby aiding discovery of R and PR genes and breeding for resistance to cotton diseases.

## Introduction

Cultivated plant species are under continuous attack by pathogens, which imposes a major challenge for growers by causing significant crop yield loss (Blasingame and Patel, 2004; Roberts et al., 2007). The future of crop improvement depends on understanding of the distribution, structure, and organization of disease resistance (R) and pathogen-induced (PR) genes (Ulloa et al., 2011). Plants have a great capacity to recognize pathogen effectors and inducers through different strategies (Dodds and Rathjen, 2010); however, our understanding of these strategies and interactions is still limited. New DNA sequence information coupled with the physical alignment of genomic regions into chromosomal maps and the anchoring of genetic maps are all steps that will improve the accuracy of detecting R or PR genes (van Loon et al., 2006; Bent and Mackey, 2007; Kou and Wang, 2010; Ulloa et al., 2011) and gene functions of important biological processes in crops (Rong et al., 2004; Ulloa et al., 2007; Chaudhary et al., 2009). In addition, these new discoveries will have important implications for breeding effective pest and disease resistance into elite cultivars by marker-assisted selection (MAS) (Ulloa et al., 2011, 2013).

Plants express multiple R genes with specificities for different strains of viruses, bacteria, fungi and nematodes, and individual plant genomes include hundreds of R gene-like sequences (Bent and Mackey, 2007; Adams-Phillips et al., 2008; Ulloa et al., 2011). The most studied R genes encode putative intra-cellular proteins with nucleotide binding sites (NBS) and leucine-rich repeat motifs (LRR), which represent the largest R gene family. NBS-LRR proteins can be subdivided in two types based on structural features of the N terminus: TIR-NBS-LRR proteins which resemble the intracellular domains of Drosophila Toll and mammalian IL-1 receptors and CC-NBS-LRR proteins which contain a coiled-coil domain (Jones and Dangl, 2006; Guo et al., 2011; Qi and Innes, 2013). Based on phylogenetic relationships, most R genes reside in clusters either as tandem duplicates on a tree or mixed clusters that contain genes from different branches of a species-wide tree (Meyers et al., 2005). Different R gene-mediated signal transduction pathways may utilize some distinct signaling components and induce a set of plant responses (Sato et al., 2007; Adams-Phillips et al., 2008). In contrast, PR genes have been classified into 17 families of pathogenesis-related proteins. These proteins are induced through the action of the signaling compounds of salicylic acid, jasmonic acid or ethylene (Fonseca et al., 2009; Panstruga et al., 2009; Stepanova and Alonso, 2009). They possess antimicrobial activities *in vitro* through hydrolytic activities on cell walls, contact toxicity, and perhaps an involvement in defense signaling. However, these proteins serve essential plant functions (senescence, wounding, cold stress, and present in floral tissue) whether they are used in defense or not (van Loon et al., 2006).

In cotton (*Gossypium* spp.), root-knot nematode [RKN (*Meloidogyne incognita*)], reniform nematode [REN (*Rotylenchulus reniformis*)], Fusarium wilt [FOV) (*Fusarium oxysporum* f.sp. *vasinfectum*)], Verticillium wilt [VW (*Verticillium dahliae*)], and black root rot [BRR (*Thielaviopsis basicola*)] represent expanding threats to crop production (Wang et al., 2006; Niu et al., 2008; Dighe et al., 2009; Ulloa et al., 2011, 2013; Fang et al., 2014; Zhao et al., 2014). Cotton is one of the most economically important crops, providing the world's leading natural fiber, and it is a polyploidy model for cytogenetic, genomic, and evolutionary biology research (Kim and Triplett, 2001; Wendel and Cronn, 2003; Ulloa et al., 2007; Chaudhary et al., 2009). The estimated cotton yield loss due to diseases was 10.93% in the United States in 2004 (Blasingame and Patel, 2004). Increased knowledge of resistance to cotton pathogens such as RKN, REN, FOV, VW, BRR, and of genomic segments housing R or PR genes will help to elucidate the mechanisms of qualitative and quantitative disease resistance.

Knowledge of R and PR genes has increased with the availability of genome data and the increasing number of genes reported to be involved in resistance (Ulloa et al., 2007). New DNA sequences can be examined to discover genes involved in disease resistance by sequence comparisons with existing databases of expressed sequence tags (ESTs) such as GenBank (http://www.ncbi.nlm.nih.gov/). Additional studies using genomic and proteomic technologies have facilitated global comparisons of R and PR expression profiles (Ulloa et al., 2011; Yin et al., 2012; Wang et al., 2013; Wei et al., 2013) and pathway components of genes involved in disease defense and/or response (Chisholm et al., 2006).

Integrating disease resistance phenotypes into high-yielding, high-fiber quality cultivars is one of the most important objectives in cotton breeding programs (Ulloa et al., 2011). To further elucidate and expedite the discovery of R and/or PR genes; herein, we provide new DNA sequence information of large genomic segments (e.g., BAC clones) from cv. Acala Maxxa (*G. hirsutum* L.) for which MUSB-derived single sequence repeat (SSR) markers were previously mapped to chromosomes (Chr) 11 and 21 (Frelichowski et al., 2006; Ulloa et al., 2008; Yu et al., 2012). These markers reportedly underlie QTLs involved in disease resistance; therefore, capturing and sequencing BAC-sized genomic segments tightly linked to these SSRs will help to resolve local content and genome structure of RKN (Shen et al., 2006; Wang et al., 2006; Ynturi et al., 2006; Ulloa et al., 2010), REN (Dighe et al., 2009; Gutiérrez et al., 2011); FOV (Ulloa et al., 2011, 2013), VW (Bolek et al., 2005; Fang et al., 2014; Zhao et al., 2014), and BRR (Niu et al., 2008) resistance. The Maxxa BAC clone and marker sequence data were also compared to the whole genome sequence assemblies of the *G. raimondii* D_5_ and *G. arboreum* A_2_ ancestral diploid genomes (Paterson et al., 2012; Wang et al., 2012b; Li et al., 2014) and domesticated tetraploid TM-1 AD_1_ (*G. hirsutum*) genome which are now publicly available (Li et al., 2015; Zhang et al., 2015).

## Materials and methods

### Selection and sequencing of BAC clones of Upland cotton chromosomes 11 and 21

Two strategies were deployed to recruit BAC clones that mapped to Upland cotton Chr 11 and Chr 21 from the cv. Acala Maxxa genomic library (Tomkins et al., 2001). The first strategy used MUSB SSR markers previously mapped to Chr 11 (Frelichowski et al., 2006). Some of these marker-loci were later placed on Chr 21 (Ulloa et al., 2008; Yu et al., 2012). We selected BAC clones which contained 12 MUSB SSRs (Table 1) from these two chromosomes. Some of these selected MUSB markers were identified as being associated with FOV resistance, using genetic and QTL mapping methods, and bulked segregant analysis (BSA) on resistant and susceptible progeny with different genetic backgrounds (Ulloa et al., 2011, 2013; *Ulloa M and Roberts P unpublished information*). Other MUSB markers were selected because they were mapped in the vicinity of an underlying QTL involved in pathogen resistance (Table 2).

**Table 1:** **Bacterial artificial chromosome (BAC) and derived MUSB SSR marker names, and number of Unigenes predicted based on G. *hirsutum* Unigene NCBI database and genes predicated based on Augustus Gene Prediction Software of BAC clones on Upland cotton chromosomes 11 and 21**.

**ID of BAC**	**Marker**	**Seq length bp**	**Contigs #**	***G. hirsutum*** **unigene**					**Augustus prediction genes**				
				**Total Uni-Genes**	**Genes matching with NR protein Blast**	**Unigenes associated with SRE**	**Transpo-sable elements**	**dna/rna polymerase proteins**	**Total Genes**	**Genes matching with NR protein Blast**	**Unigenes associated with SRE**	**Transpo-sable elements**	**dna/rna polymerase proteins**
**Chr11**													
28E08	MUSB1000	117,929	5	97	72	13	14	28	20	13	8	3	3
28O10	MUSB1015	135,685	10	98	19	1	1	15	12	10	3	3	3
26K03	MUSB0953	136,740	4	42	27	1	10	15	13	10	4	5	1
24E04	MUSB0641	107,318	1	27	16	0	7	0	18	15	0	7	1
40I16	MUSB1278	89,982	5	32	15	1	1	12	7	6	3	3	0
34K01	MUSB1163	136,264	13	66	26	2	9	14	17	13	5	6	2
29O06	MUSB1035	111,818	5	10	4	0	1	0	16	15	2	11	1
33K23	MUSB0827	105,004	5	61	39	3	1	36	10	9	3	3	4
18O18	MUSB0404	75,523	10	8	2	2	0	0	14	14	7	7	0
31K15	MUSB1076	113,182	9	26	18	18	0	0	19	19	8	8	0
		1,129,445	67	467	238	41	44	120	146	124	43	56	15
**Chr21**													
AC193383^*^	NAU6334 NAU6598 NAU6673 NAU6301	107,036	3	9	8	8	0	0	16	14	13	0	0
AC187848	NAU2826 NAU6222 NAU6627	88,499	2	26	17	17	0	0	13	10	9	2	0
30E04	MUSB0810	112,602	10	71	67	58	9	0	8	8	5	1	0
AC187214	NAU2110 NAU6178 NAU6444	99,687	2	10	6	6	0	0	8	5	1	3	0
32H19	MUSB0823	87,789	2	19	6	6	0	0	3	2	1	0	0
AC187470^**^	NAU6224 NAU6282 NAU1063	110,475	1	62	57	57	0	0	6	5	2	1	0
AC202821	NAU6146 NAU6677	88,339	7	14	8	8	0	0	6	4	3	1	0
AC190836	TMB1871 NAU6507	85,559	6	1	1	1	0	0	9	8	5	3	0
AC202830	NAU6520 NAU6697 NAU6530 NAU6658 NAU6675 NAU6593	101,659	4	32	31	31	0	0	13	12	11	1	0
AC187810	NAU6525 NAU6431 NAU6245	92,907	1	41	32	32	0	0	16	16	9	4	0
		974,552	38	285	233	224	9	0	98	84	59	16	0

* AC193383: NAU6334, NAU6598, NAU6301, mapped to both chr 11 and chr 21; NAU6673, mapped to Chr 11.

** *AC187470: NAU6224, NAU6282 mapped to Chr 21, NAU1063 mapped to both Chr 11 and Chr 21*.

**Table 2:** **SSR markers underlying QTL associations with nematode and pathogen resistance genes on Upland cotton chromosomes 11 and 21**.

**Resistance gene**	**Nematode or pathogen**	**Closest marker identified**		**References**
		**Chr 11**	**Chr 21**	
RKN	Root-knot nematode	CIR316		Wang et al., 2006, 2012a; Ynturi et al., 2006; Gutiérrez et al., 2010; Ulloa et al., 2010
		MUCS088		Wang et al., 2008
		BNL1231	BNL1231	Bezawada et al., 2003; Wang et al., 2012a
Ren	Reniform nematode	BNL3279	BNL3279	Robinson et al., 2007; Dighe et al., 2009; Romano et al., 2009; Gutiérrez et al., 2011
			Gh132	Gutiérrez et al., 2011
FOV	Fusarium Wilt	MUSB0827 MUCS399 MUSB1015		Ulloa et al., 2011
			MUSB0823	Ulloa et al., 2011, 2013 (Unpublished data)
VW	Verticillium Wilt			Bolek et al., 2005
		DPL0500a-DPL0522 Not included in Figure 1	TMB1637-DPL0500b	Fang et al., 2014 (no marker on Figure 1)
		NAU5428		Zhao et al., 2014
BRR	Black root rot	BNL3442-BNL1034		Niu et al., 2008

The second strategy was to use SSR marker-sequences previously mapped on Chr 11 and Chr 21 (CMD: http://www.cottonmarker.org/) to select BAC clones previously sequenced from the Acala Maxxa library by sequence-comparison. These BAC clones were originally sequenced erroneously as part of the maize sequencing project by the Genome Sequencing Center, Washington University School of Medicine. The DNA sequence information of these BACs was deposited into GenBank under the accession numbers: AC193383, AC187848, AC187214, AC187470, AC202821, AC190836, AC202830, and AC187810. Sequences of each BAC clone (Table 1) were compared to SSR marker-sequences from Chr 11 and Chr 21. The selection criteria of tagging a BAC clone with mapped SSR markers from these chromosomes were as follows: only the sequence of each SSR marker spanning forward primer to the reverse primer (including the SSR motif) was used for the comparison. DNA sequences were blasted using all six frames (forward +1 to +3 and reverse −1 to −3) base positions. Potential BAC clones were tagged with an SSR marker when both (BAC and SSR) DNA sequences had a similarity >96%.

### Sequencing and assembly of Upland cotton BAC clones

A small-insert (3–5 kb) library was constructed from each of the 12 BAC clones, which harbored the selected MUSB markers on Chr 11 and Chr 21 (Table 1). Small-insert DNA fragments were generated by isolating BAC DNA as a maxi-prep from the BAC clone and subjecting the DNA to random fragmentation by hydroshearing (Digilab®, Digilab Inc., Holliston, MA). Fragments between 3 and 5 kb were size-selected by gel electrophoresis, were end-repaired and cloned into the hi-copy plasmid-based cloning vector pBlueskriptKSII+ (Agilent Technologies) and then electroporated into *E. coli* DH10B host cells. Transformants were selected on Lysogeny broth (LB) plates containing carbenicillan, X-Gal and IPTG. White recombinant colonies were picked robotically using the Genetix Q-bot (Genetix, Boston, MA) and stored as individual clones in Genetix 96-well microtiter plates as glycerol stocks at −80°C. Sequencing was performed using the Dye-terminator cycle sequencing kit v3.1 (Applied Biosystems, Foster City, CA). Sequence data from the forward and reverse universal priming sites of the shotgun clones were accumulated on an ABI 3730xl DNA analyzer (Applied Biosystems, Foster City, CA). The BAC clones were sequenced to approximately 8X clone coverage (assuming 120 kb average insert size) and assembled with PHRAP software (Ewing et al., 1998), and edited with Consed (Gordon et al., 1998). Sequence contigs were ordered and oriented by the bridging shotgun method, and gaps were joined by the addition of N's giving a single contiguous consensus sequence for analysis. The sequencing of the BAC clones, which harbored the MUSB markers, was performed at Clemson University Genomics Institute, SC, USA. Additional information about the sequencing of these clones can be found in Ulloa et al. (2011). The DNA sequence information of these BACs was deposited into GenBank under the accession numbers: KM396694 (28E08), KM396695 (28O10), KM396696 (26K03), KM396697 (24E04), KM396698 (40I16), KM396699 (34K01), KM396700 (29O06) KM396701 (33K23), KM396702 (18O18), KM396703 (31K15), KM396704 (30E04), and KM396705 (32H19). The numbers and letters identify the BAC clone.

### BAC sequence annotation of stress response elements

DNA sequence-local alignments were made with the comprehensive *G. hirsutum* unigene set from http://www.plantgdb.org. The Unigene set consisting of 98,420 Unigenes (*G. hirsutum* mRNA assembly May 8, 2008; based on GenBank release 165) was downloaded from PlantGDB (www.plantgdb.org). Unigene sequences were BLASTN aligned to each BAC sequence individually with an *e* ≤ 1e-5 and identity ≥90%. Gene Ontology (GO) annotation was conducted using the Blast2GO program with default parameters (Gene Ontology Consortium, 2006; Conesa and Gotz, 2008). Gene prediction and annotation were performed using the prediction program Augustus (Stanke and Morgenstern, 2005). The Augustus program was tested on the *Arabidopsis* gene set, which considers expressed sequence tag (EST) matches as additional support for gene identification. All predicted genes and unigenes were subjected to a similar analysis using BLASTX through the National Center for Biotechnology Information (NCBI) (http://www.ncbi.nlm.nih.gov/) nr protein database with a value of 1e-5 to identify previously established protein motifs. Stress response elements (SRE) were identified based on the description of bioprocess of GO annotation. Genes involved in stress response elements were identified according to associated protein molecular function (MF), bioprocess (BP), and cell component (CC).

### Alignment to *Gossypium raimondii* (D_5_), *G. arboreum* (A_2_), *G. hirsutum* TM-1 (AD_1_), and other genomes

BAC sequences were aligned to the *G. raimondii* diploid D_5_ whole genome (phytozome.net) (Paterson et al., 2012) through NCBI-nucleotide BLAST, *G. arboreum* diploid A_2_ whole genome (http://cgp.genomics.org.cn) (Li et al., 2014) and TM-1 AD_1_ genome (http://cottongen.org) from two independent groups (CGP-BGI group, Li et al., 2015; NAU-NBI group, Zhang et al., 2015) with an *e* ≤ 1e-10 and identity ≥90%. The comparisons of the BAC sequences on Chr 11 and Chr 21 with corresponding chromosomes in A_2_, D_5_, AD_1_ genome backgrounds were conducted. The average identity and the percentage of mapped BAC sequences were calculated based on consecutive matched sequence with compared genomes. The TM-1 sequence from the CGP-BGI group was used as a genome background to determine that resistance genes from these BACs are more frequently located in the regions of Chr 11 and Chr 21 with Fisher's exact test (*P* < 0.05). Comparisons were also made between these BACs and other plant taxa: *Arabidopsis thaliana, Vitis vinifera, Populus trichocarpa*, and *Theobroma cacao*.

### Selection of SSR markers and construction of linkage groups

We targeted all SSR markers previously mapped on Upland cotton Chr 11 and Chr 21 (CMD: http://www.cottonmarker.org/), especially those underlying QTLs determining resistance to RKN (Shen et al., 2006; Wang et al., 2006, 2012a; Ynturi et al., 2006; Gutiérrez et al., 2010; Ulloa et al., 2010), REN (Dighe et al., 2009; Romano et al., 2009; Gutiérrez et al., 2011), FOV (Ulloa et al., 2011, 2013), VW (Bolek et al., 2005; Fang et al., 2014), and BRR (Niu et al., 2008). QTL analyses of marker-resistance associations for RKN, REN, FOV, VW, and BRR on these chromosomes were reported from previous publications (Table 2).

**Figure 1:**
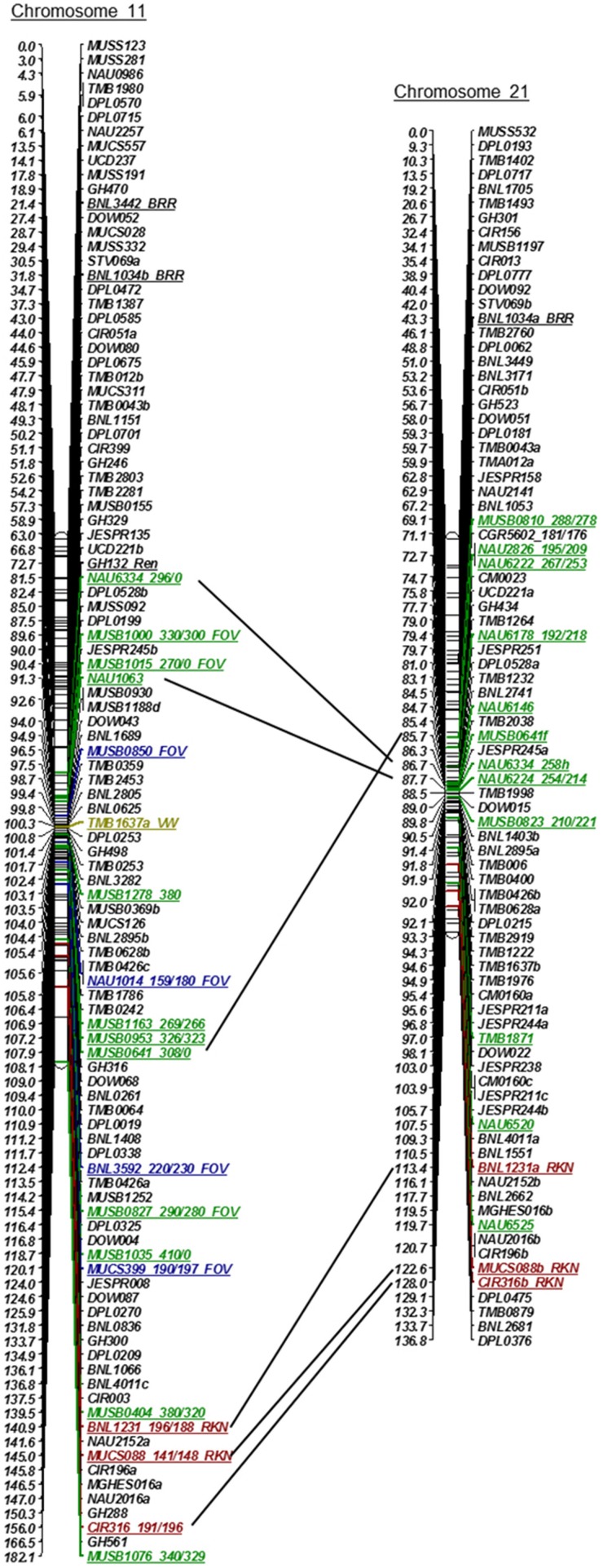
**Linkage maps of Chr 11 and its homoeologous Chr 21 using an interspecific [Upland TM-1 (*Gossypium hirsutum*) x Pima 3-79 (*G. barbadense*)] RIL population (Yu et al., 2012), showing relationships between molecular markers and underlying QTLs involved in resistance to root-knot nematode (RKN, Shen et al., 2006; Wang et al., 2006; Ynturi et al., 2006; Ulloa et al., 2010), reniform nematode (REN, Dighe et al., 2009; Gutiérrez et al., 2011); Fusarium wilt (FOV, Ulloa et al., 2011), Verticillium wilt (VW, Fang et al., 2014; Zhao et al., 2014), and black root rot (BRR, Niu et al., 2008)**.

Initially, 1100 SSR markers (BNL, CIR, GH, MUSB, MUCS, MUSS, NAU, DPL, DOW, and TMB) were used with wide coverage to construct the linkage groups of Chr 11 and Chr 21 on the recombinant inbred line (RIL) population of Upland TM- 1 × Pima 3-79 (Frelichowski et al., 2006; Ulloa et al., 2008, 2011, 2013; Wang et al., 2012a; Yu et al., 2012). Additional SSR markers identified to be tagged to a BAC clone or clones were mapped using JoinMap^R^ version 4.0 (Van Ooijen, 2006). Likelihood ratio (LOD) scores of 8–12 were examined for each linkage group/chromosome using the Kosambi mapping function and a maximum distance of 40 cM on this population. Moreover, using the anchored SSR markers (MUSB) of these linkage groups and their recombination frequencies or cM distances, SSR markers were placed on Chr 11 and Chr 21 linkage groups (Figure 1) on the most recent published linkage maps of the TM-1 x 3-79 RIL population (Yu et al., 2012). Only the name of SSR markers was included in Figure 1, keeping their original cM distance between the SSR markers.

### Marker analysis and data mining

SSR markers previously mapped on Chr 11 and Chr 21 reported in the Cotton Marker Database (CMD: www.cottonmarker.org) were used to investigate DNA sequence composition. Sequences were then BLASTed through the NCBI (http://www.ncbi.nlm.nih.gov/). Sequences were compared against three databases: (a) Nucleotide collection (nr/nt); (b) Expressed Sequence Tags (EST); and (c) Non-Redundant protein sequences (nr). The top sequence hits found for each sequence in all three databases were then BLASTed through GO (http://www.geneontology.org/). The top functional hits given by GO were collected along with their categorized gene products [biological process (BP), cellular component (CC), and molecular function (MF)]. SSR markers involved in defense response or stress response were categorized according to top blasted protein function (receptor, disease protein, transcription factor, and oxygen-reduction and so on) and GO annotation.

## Results

### BAC sequence and annotation for stress response elements

Twenty selected BAC clones were analyzed for potential coding elements involved in response to biotic/abiotic stress mechanisms (Table 1). Twelve BAC clones tagged with BAC-end MUSB [selected from Frelichowski et al. (2006) and Ulloa et al. (2008, 2011)] markers were sequenced: BAC-derived MUSB0404, MUSB0641, MUSB0827, MUSB0953, MUSB1000, MUSB1015, MUSB1035, MUSB1076, MUSB1163, and MUSB1278 from Chr 11, and MUSB0810 and MUSB0823 from Chr 21 (Table 1). The estimated BAC clone size according to assembled sequence data ranged from 68 to 140 kb with an average of 106 kb per BAC. The BAC clones were sequenced to an approximate 8X coverage, which resulted in 3–8 ordered contigs spanning up to 140,000 bp. In addition, seven BAC clones tagged to previously mapped SSR markers (25 NAUs and one TMB) on Chr 21 from the Upland cotton cultivar Acala Maxxa genomic library previously sequenced by the Genome Sequencing Center, Washington University School of Medicine were also investigated for potential coding elements: AC193383, AC187848, AC187214, AC187470, AC202821, AC190836, AC202830, and AC187810 (Table 1). These Maxxa BACs, erroneously sequenced by the maize group, were used in a different cotton characterization study by Guo et al. (2008). In this study, the 10 BAC clones from Chr 11 yielded a total of 1,129,445 bp while the 10 BAC clones from Chr 21 yielded 974,552 bp, for a total of 2,103,997 bp sequence data.

BAC sequence annotation by BLASTN alignment to the publicly available *G. hirsutum* Unigene set (GenBank release 165) revealed 467 (Chr 11) and 285 (Chr 21) putative Unigenes (*e* ≤ 1e-5). Functional signature annotations of BAC-mapped Unigene sequences were aligned to the non-redundant protein database and assigned GO terms. A total of 238 out of 467 of Chr 11 and 233 out of 285 of Chr 21 putative Unigenes were found to be similar to known protein sequences with *e* ≤ 1e-5 (Table 1), while 229 putative Unigenes on Chr 11 and 52 on Chr 21 had no match to known protein sequences with *e* ≤ 1e-5 (Table 1 and Tables S1, S2). There were 41 Unigenes on Chr 11 and 224 on Chr 21 involved in disease defense response or stress response elements (SRE) (Table 1) based on sequence description from the BLASTed protein database and GO annotations [P (bioprocess), F (molecular function) and C (cell component)] (additional information highlighted in yellow in Tables S1, S2). Stress response elements involved in internal and external stimulus, stress, signaling process and cell death from these Unigenes are shown in Table S3 for Chr 11 and Table S4 for Chr 21. In addition, 44 transposable elements (TEs) and 120 DNA/RNA polymerase family proteins were identified on Chr 11, and nine TEs but no DNA/RNA polymerase protein on Chr 21 (Table 1).

Augustus gene prediction software revealed 146 genes on Chr 11 and 98 genes on Chr 21. The results indicated abundance of genes with considerable homology to disease response elements for these BAC clones (Table 1 and Tables S5–S8), with function in cellular growth and development processes, transport, translation, plus metabolic functions and stress response elements. Forty-three genes on Chr 11 BACs and 59 genes on Chr 21 BACs were involved in defense response (Table 1 and Tables S5, S6 highlighted in yellow), including receptor kinase proteins, early-responsive to dehydration stress proteins, subtilisin-like serine endopeptidase family proteins, strictosidine synthase-like, universal stress proteins, auxin-responsive proteins, and disease resistance proteins involved in stress response. GO annotation showed a range of defense associated proteins for MF, and SRE included responses to biotic/abiotic stimulus, signaling, and cell death (Tables S7, S8).

The Augustus gene prediction software also indicated 56 TE on Chr 11 BACs and 16 on Chr 21 BACs (Table 1). TE included retrotransposon ty1-copia subclass, retrotransposon ty3-gypsy subclass, gag-pol polyprotein, mutant gag-pol polyprotein, mutator sub-class protein and copia-like retrotransposable elements (Table 3, Tables S5, S6). The longest TE hit length extended 6759 bp. A GO analysis further characterized these TE into a range of defense-related acitivities (Table 3 and Tables S5, S6). In addition to the TEs, 15 DNA/RNA polymerase family proteins were identified on Chr 11 but none were identified on Chr 21 (Table 1).

**Table 3:** **BAC sequences of Upland cotton chromosomes 11 and 21 that contain disease resistance encoded protein annotation with associated transposable elements**.

**Sequence name**	**Sequence description**	**Sequence length**	**Hit ACC**	**Mean similarity**	**#GOs**	**GOs**
31K15:g127.t1	Gag-pro-like protein	555	EOY21122	78.15%	0	–
31K15:g128.t1	Uncharacterized protein tcm_003795	1368	EOX94247	49.45%	0	–
31K15:g129.t1	Gag-pol polyprotein	2775	ABO36622	55.70%	6	F:nucleic acid binding; P:DNA integration; F:zinc ion binding; F:exonuclease activity; C:intracellular; F:metal ion binding
31K15:g130.t1	nbs-containing resistance-like protein	411	CAN83754	54.20%	4	P:defense response; F:ADP binding; F:hydrolase activity; F:phosphoprotein phosphatase activity
31K15:g131.t1	Retrotransposon unclassified	3288	CAN59755	60.80%	3	F:nucleic acid binding; P:DNA integration; F:zinc ion binding
31K15:g132.t1	cc-nbs-lrr resistance protein	594	XP_002526758	55.75%	1	F:hydrolase activity
31K15:g133.t1	Leucine-rich repeat containing protein	1338	AAO37645	48.25%	4	P:defense response; F:ADP binding; F:hydrolase activity; F:phosphoprotein phosphatase activity
31K15:g134.t1	Retrotransposon unclassified	4848	EOY11267	63.10%	2	F:organic cyclic compound binding; F:heterocyclic compound binding
31K15:g135.t1	Copia-type polyprotein	4968	EOY11267	64.50%	3	F:heterocyclic compound binding; F:organic cyclic compound binding; P:cellular process
31K15:g136.t1	Vamp protein sec22	633	EOY08448	76.70%	3	P:defense response to virus; C:plasmodesma; C:plasma membrane
31K15:g137.t1	Polyprotein	3426	CAN81099	63.45%	7	P:electron transport chain; P:DNA recombination; C:plastid; F:electron carrier activity; F:DNA binding; F:ion binding; P:electron transport
31K15:g137.t2	Polyprotein	3411	CAN81099	63.45%	7	P:electron transport chain; P:DNA recombination; C:plastid; F:electron carrier activity; F:DNA binding; F:ion binding; P:electron transport
31K15:g138.t1	Uncharacterized protein partial(retropepsin-like protein)	2544	AFN88198	47.95%	6	F:RNA binding; F:nucleic acid binding; P:DNA integration; P:RNA-dependent DNA replication; F:zinc ion binding; F:RNA-directed DNA polymerase activity
31K15:g139.t1	Disease resistance protein	1797	CAN74029	73.55%	10	P:response to stimulus; F:purine ribonucleoside binding; P:single-organism cellular process; F:anion binding; F:zinc ion binding; F:nucleic acid binding; P:ammonium transport; P:DNA integration; C:membrane; F:adenyl ribonucleotide binding
31K15:g140.t1	Copia-like retrotransposable	1356	EOY19734	49.20%	3	F:nucleic acid binding; P:DNA integration; F:zinc ion binding
31K15:g141.t1	Mitogen-activated protein kinase kinasekinase	522	EOY25694	59.40%	1	F:transferase activity, transferring phosphorus-containing groups
31K15:g142.t1	nbs-containing resistance-like protein	906	XP_003598563	65.80%	4	P:defense response; F:ADP binding; F:hydrolase activity; F:phosphoprotein phosphatase activity
31K15:g143.t1	Copia-like retrotransposable	3780	EOY11267	62.20%	3	F:heterocyclic compound binding; F:organic cyclic compound binding; P:cellular process
31K15:g144.t1	nbs-lrr resistance protein rgh2	1470	AAO37645	59.40%	1	F:hydrolase activity
AC190836:g35.t1	cc-nbs-lrr class disease resistance	1308	EOY13112	65.15%	2	P:defense response; F:ADP binding
AC190836:g36.t1	cc-nbs-lrr class disease resistance protein	1737	EOY13110	62.35%	7	P:defense response; F:ADP binding; F:hydrolase activity; F:phosphoprotein phosphatase activity; F:ATP binding; F:nucleotide binding; F:nucleoside-triphosphatase activity
AC190836:g37.t1	Gag-pol polyprotein	1758	CAN64972	71.80%	1	F:binding
AC190836:g38.t1	cc-nbs-lrr class disease resistance	543	EOY13112	70.63%	0	–
AC190836:g39.t1	Retrotransposon unclassified	1848	AAG50698	66.95%	1	F:binding
AC190836:g40.t1	Retrotransposon ty1-copia subclass	3408	AAM91886	61.90%	2	P:DNA recombination; F:DNA binding
AC190836:g42.t1	Leucine-rich repeat protein kinase family	906	EOY13449	63.30%	0	–
AC190836:g43.t1	cc-nbs-lrr class disease resistance	852	EOY13112	64.20%	2	P:defense response; F:ADP binding
AC202830:g44.t1	tmv resistance protein	2512	EOY25754	68.00%	1	F:nucleotide binding
AC202830:g45.t1	Disease resistance	768	EOY25754	52.85%	6	P:defense response; F:ADP binding; P:signal transduction; F:ATP binding; F:nucleotide binding; F:nucleoside-triphosphatase activity
AC202830:g46.t1	Serine threonine-protein phosphatase 7 long form homolog	333	XP_004490229	69.40%	2	F:nucleic acid binding; F:zinc ion binding
AC202830:g48.t1	Probable adp-ribosylation factor gtpase-activating protein agd14-like	2676	EOY10412	67.30%	2	F:metal ion binding; P:nucleobase-containing compound metabolic process
AC202830:g48.t2	gtpase activating isoform 4	2553	EOY10413	72.00%	4	F:nucleoside-triphosphatase activity; F:ion binding; P:nucleobase-containing compound metabolic process; F:nucleotide binding
AC202830:g49.t1	tmv resistance protein	2553	EOY25754	69.85%	1	F:nucleotide binding
AC202830:g49.t2	tmv resistance protein	2991	EOY25754	69.40%	1	F:nucleotide binding
AC202830:g49.t3	tmv resistance protein	5583	EOY25754	69.25%	1	F:nucleotide binding
AC202830:g50.t1	Strong similarity to gi	2205	CAN81099	58.65%	5	F:nucleic acid binding; P:DNA integration; F:zinc ion binding; P:oxidation-reduction process; F:oxidoreductase activity
AC202830:g51.t1	Retrotransposon ty1-copia sub-class	1368	CAN83392	70.05%	3	F:DNA binding; C:plastid; P:DNA recombination
AC202830:g52.t1	Cysteine-rich rlk (receptor-like protein kinase) 8	594	CAN75536	76.00%	7	F:DNA binding; F:peroxidase activity; F:zinc ion binding; P:DNA integration; P:DNA recombination; P:peroxidase reaction; P:response to oxidative stress
AC202830:g53.t1	chromo domain-containing protein lhp1-like	581	EOY10373	62.35%	5	P:single-organism cellular process; P:cellular macromolecule metabolic process; P:negative regulation of gene expression; P:primary metabolic process; P:regulation of gene expression, epigenetic
AC187810:g54.t1	t-complex protein 1 subunit theta-like	564	EOY11214	84.05%	16	P:cytoskeleton organization; P:gluconeogenesis; P:protein folding; C:membrane; P:pyrimidine ribonucleotide biosynthetic process; P:RNA methylation; P:proteasomal protein catabolic process; P:cullin deneddylation; P:photomorphogenesis; C:cytosol; P:G2 phase of mitotic cell cycle; F:unfolded protein binding; C:plasmodesma; P:regulation of flower development; P:histone lysine methylation; F:ATP binding
AC187810:g54.t2	t-complex protein 1 subunit theta-like	663	EOY11214	85.10%	16	P:cytoskeleton organization; P:gluconeogenesis; P:protein folding; C:membrane; P:pyrimidine ribonucleotide biosynthetic process; P:RNA methylation; P:proteasomal protein catabolic process; P:cullin deneddylation; P:photomorphogenesis; C:cytosol; P:G2 phase of mitotic cell cycle; F:unfolded protein binding; C:plasmodesma; P:regulation of flower development; P:histone lysine methylation; F:ATP binding
AC187810:g55.t1	Disease resistance	2697	EOY25762	64.05%	1	F:binding
AC187810:g56.t1	Uncharacterized protein tcm_026511	2757	CAN74029	70.20%	2	F:binding; P:cellular process
AC187810:g57.t1	Retrotransposon ty1-copia subclass	303	CAN71828	76.50%	5	F:DNA binding; F:zinc ion binding; P:DNA integration; P:DNA recombination; C:mitochondrion
AC187810:g58.t1	Disease resistance	2370	EOY25754	64.35%	3	P:defense response; F:ADP binding; P:signal transduction
AC187810:g58.t2	Disease resistance protein	1695	ERP57954	56.50%	3	P:defense response; F:ADP binding; P:signal transduction
AC187810:g59.t1	Retrotransposon unclassified	2748	EOY32548	57.45%	1	F:binding
AC187810:g59.t2	Retrotransposon unclassified	2226	CAN59755	58.70%	3	F:nucleic acid binding; P:DNA integration; F:zinc ion binding
AC187810:g60.t1	tmv resistance protein	2316	EOY25760	65.15%	6	P:defense response; F:ADP binding; P:signal transduction; F:ATP binding; F:nucleotide binding; F:nucleoside-triphosphatase activity
AC187810:g60.t2	tmv resistance protein	2439	EOY25760	65.10%	6	P:defense response; F:ADP binding; P:signal transduction; F:ATP binding; F:nucleotide binding; F:nucleoside-triphosphatase activity
AC187810:g61.t1	tmv resistance protein	1827	EOY25760	66.90%	1	F:nucleotide binding
AC187810:g61.t2	Disease resistance	2334	EOY25754	62.00%	3	P:defense response; F:ADP binding; P:signal transduction
AC187810:g62.t1	Uncharacterized protein tcm_026511	3936	EOY11267	63.30%	2	F:organic cyclic compound binding; F:heterocyclic compound binding
AC187810:g63.t1	Retrotransposon unclassified	2271	CAN64779	66.05%	4	F:transition metal ion binding; F:heterocyclic compound binding; P:nucleobase-containing compound metabolic process; F:organic cyclic compound binding
AC187810:g64.t1	arm repeat protein interacting with abf2-like	498	XP_003522041	79.20%	3	F:nucleic acid binding; F:zinc ion binding; P:DNA integration

*Yellow color means disease resistance protein and blue color means disease receptor protein and transposable elements (TE)*.

Twenty-three disease resistance proteins were identified in four BACs (31K15 on Chr 11, and AC190836, AC202830 and AC187810 on Chr 21). The BAC 31K15 associated with marker MUSB1076 linked to R gene *rkn1* (Wang et al., 2006) and cluster regions containing leucine-rich repeat protein, NBS-LRR resistance protein *rgh2* or *rgh1*, and CC-NBS-LRR resistance protein. Three BAC clones (AC190836, AC202830, and AC187810) on Chr 21 contained R genes harboring NBS-LRR proteins, including CC-NBS-LRR class disease resistance, tmv resistance protein and other disease resistance proteins (Table 3). Based on structural features of the N terminus, NBS-LRR proteins were surrounded by additional receptor proteins such as serine-threonine and kinase-like proteins, and TEs (Table 3). Moreover, NBS-LRR genes were identified within clusters and in the vicinity of the RKN, REN, FOV, VW, and BRR resistance of marker-genes previously reported (Bolek et al., 2005; Wang et al., 2006; Niu et al., 2008; Dighe et al., 2009; Ulloa et al., 2011).

**Figure 2:**
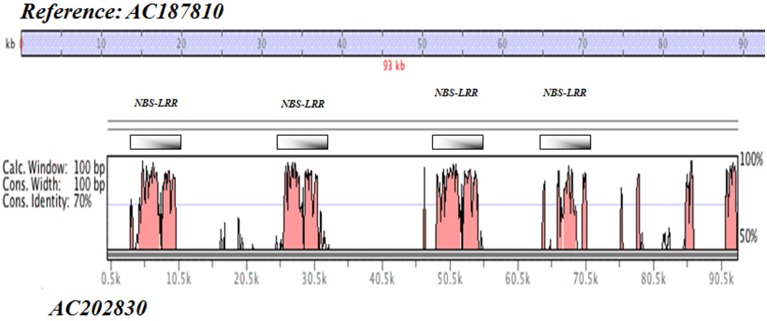
**Self-alignment of BAC clones in Upland cotton Chr 21**. Percent identity plot of duplication harboring NBS-LRR resistance motifs (BAC clones AC187810 vs. AC202830 on Chr 21).

More specifically, a percent identity plot of duplication harboring NBS-LRR resistance motifs for BAC clones AC187810 vs. AC202830 on Chr 21 is given in Figure 2, in which a set of seven regions were found harboring NBS-LRR motifs with a minimum of 70% identity spanning the clone length of ~90 kb.

### Alignment to *Gossypium raimondii* (D_5_), *G. arboreum* (A_2_), *G. hirsutum* TM-1 (AD_1_) and other genomes

A synteny block comparison was made of alignment of full length sequences of Chr 11 and 21 BAC clones to the two available assembled whole diploid genome sequences of *G. arboreum* (A_2_) and *G. raimondii* (D_5_) (Tables S9–S13). The comparisons among the matched sequences showed 84.23% identity with Chr 11 BACs and 98.54% identity with BACs of Chr 21 of the tetraploid (AD) genome, corresponding to D_5_ Chr 7 genome sequence (Tables S9, S12, S13). Eight percent and 80% consecutive sequences from chromosomes 11 and 21, respectively were mapped to D_5_ Chr 7. Seven Chr 11 BACs with no consecutive mapping sequence were also mapped to D_5_ Chr 7 in several regions. Most matched sequences of these seven BACs were TEs (Tables S9, S12, S13) which showed multiple copies through the whole genome, including D_5_ Chr 7. More BLAST hits of Chr 11 BACs than Chr 21 BACs with Chr 7 A_2_ genome sequence were found (Tables S9–S11). However, only one Chr 11 BAC (29O06) showed consecutive sequence length with Chr 7 A_2_ genome (Tables S9–S11). The BAC sequences matched with the A_2_ genome were mostly transposable elements which are distributed across the whole genome.

Alignment of Chr 11 and Chr 21 BAC sequences from *G. hirsutum* Maxxa to *G. hirsutum* TM-1 genome showed slight differences between the two sequencing groups BGI and NBI, possibly due to different assembly methods (Tables S14, S15). In total, 42 and 52% consecutive sequences of Maxxa BACs on chromosomes 11 and 21, respectively, were mapped to TM-1 At-Chr1 (equals Chr 11) and Dt-Chr7 (equals Chr 21) from BGI sequencing data (Tables S14, S15). From NBI sequencing data, 41 and 62% consecutive sequences of Maxxa BACs on chromosomes 11 and 21 were mapped to A11 (equals Chr 11) and D11 (equals Chr 21) of the TM-1 genome, respectively. The identities of matched sequences between Maxxa BACs and TM-1 genome reached 98% for Chr11 comparison and 97% for Chr 21 comparison with both BGI and NBI sequencing data. Some BAC sequences were aligned to unmapped scaffolds and mapped chromosomes, such as 34K01, indicating the unmapped scaffolds might be connected to the mapped chromosome. Partial consecutive sequences of the Maxxa BAC 32H19 on Chr 21 linked to the marker MUSB0823 were mapped to TM-1 genome Chr 11 (Tables S14, S15). Part of Maxxa BAC 40I16 sequence linked to MUSB1278 was mapped to Chr 7 in the TM-1 genome (Tables S14, 15). Most unmapped Maxxa BAC sequences matched with Chr 11 or Chr 21 were transposable elements across the whole genome. The enrichment analysis with Fisher's exact test indicated that 115 out of 168 GOs compared with TM-1 genome sequence from CGP-BGI group were over-represented in Chr 11 and Chr 21 regions with *p* < 0.05 (range from 8.12E-33 to 0.041). The 115 GOs included stress response elements, such as oxidoreductase activity, cell-cell signaling, defense response to virus, syncytium formation, response to abiotic stimulus, MAP kinase kinase kinase activity, and transmembrane receptor protein tyrosine kinase signaling pathway.

Comparison of Chr 11 and Chr 21 BAC sequences with four other plant taxa—*Arabidopsis thaliana, Vitis vinifera, Populus trichocarpa*, and *Theobroma cacao*, revealed conserved regions of short sequences with each plant species. Alignments with *T. cacao* and *V. vinifera* were especially strong for certain cotton BAC clones, but less so with *A. thaliana* and *P. trichocarpa*. Results from these comparisons and subsequent GO analyses did not provide additional information.

### Genetic mapping and SSR marker sequence composition

Initially, 1100 SSR markers that provided genome-wide coverage (Park et al., 2005; Frelichowski et al., 2006; Wang et al., 2006; Ulloa et al., 2008, 2011, 2013; CMD, www.cottonmarker.org) were used to develop Upland cotton Chr 11 and Chr 21 linkage groups. Matrix genotypic data of these SSR markers were used to develop the most recent genetic linkage map of the TM-1 x 3-79 RIL population (Yu et al., 2012). In addition, QTL analyses were previously conducted on Fusarium wilt phenotypic data (Ulloa et al., 2011, 2013) and root-knot nematode root-galling and egg production phenotypic data (Wang et al., 2006, 2008, 2012a; Ulloa et al., 2010) using the SSRs and related RIL populations. SSR markers associated with FOV and RKN resistance on the TM-1 x 3-79 genetic map are presented in Figure 1 (Ulloa et al., 2011, 2013; Wang et al., 2012a). SSR marker associations with resistance to RKN (Bezawada et al., 2003; Shen et al., 2006; Ynturi et al., 2006) and to other pathogens [REN (Robinson et al., 2007; Dighe et al., 2009; Romano et al., 2009; Gutiérrez et al., 2011); VW (Bolek et al., 2005; Fang et al., 2014; Zhao et al., 2014), and BRR (Niu et al., 2008)] reported by other research groups are also presented in Figure 1. The locations of the MUSB markers derived from the Acala Maxxa BAC clones (Table 1) are shown in Figure 1.

### SSR marker sequence annotation for stress response elements

Comparison of available sequence information from 256 SSRs on Chr 11 and 239 on Chr 21 to sequences in NCBI EST databases indicated considerable sequence similarity to known genes in plants, with 145 and 142 gene-homologies, respectively, of which 99 on Chr 11 and 109 on Chr 21 were indicated to play a role in plant defense. SSR sequences were similar to transcription factors R_2_R_3_-myb transcription factor, heat shock transcription factor, receptor kinase protein, light-regulated protein, zinc finger protein, leucine-rich repeat family protein, nucleic binding protein, WRKY DNA-binding protein, and Verticillium wilt resistance-like protein (Tables S16, S17). Because of duplicated loci from a single marker mapped on Chr 11 and its homoeolog Chr 21, similar genes, pseudogenes, or gene-forms may be present on both chromosomes (Figure 1; www.cottonmarker.org). Categorization of the gene function revealed that markers of Chrs 11 and 21 mapped to genes associated with all three GO: BP, CC, and MF (Tables S16, S17). GO also revealed similarities to SRE genes involved in internal and external stimulus, stress, signaling process and cell death (Table 4, Tables S18, S19). The table S20 provides data on the distance between the mapped chromosome-wide and BAC-specific markers and the defense gene sequences found on Chrs 11 and 21 listed in Table 3.

**Table 4:** **Gene ontology of marker sequences in Upland cotton chromosomes 11 and 21 that show stress response related annotations**.

**GO ID**	**Term**	**No. of Chr 11 Seqs**	**Chr 11 markers**	**No. of Chr21 seqs**	**Chr21 markers**
GO:0050896	Response to stimulus	23	NAU1063, NAU3409, NAU3493, HAU1756, NAU1148, NAU3811, NAU2877, DPL0715, NAU2809, MUSS281, NAU2257, NAU3008, NAU980, NAU3234, HAU3249, NAU5064, NAU3748, NAU5192, HAU2624, MUCS530, MUSB850, NAU2661, MON_CGR6766	21	HAU1794, MUCS347, NAU3493, HAU1805, Gh434, TMB2038, HAU0720, BNL2681, NAU2877, HAU3342, NAU3748, NAU1366, HAU2026, HAU3303, MON_DPL0582, HAU1311, MUSB850, NAU2361, NAU2758, NAU3091, NAU3895
GO:0006950	Response to stress	15	NAU1063, DPL0715, NAU2809, MUSS281, NAU2257, NAU3008, NAU980, NAU3234, HAU3249, NAU5064, MUSB850, NAU2661, NAU3409, NAU3811, MON_CGR6766	10	HAU1794, MUCS347, HAU3342, HAU1805, Gh434, TMB2038, HAU3303, MON_DPL0582, HAU1311, MUSB850
GO:0042221	Response to chemical stimulus	13	NAU3409, NAU3493, HAU1756, NAU1148, NAU3811, NAU2877, MUSS281, NAU2257, NAU3008, NAU980, NAU5192, NAU3234, MUCS530	7	NAU3493, HAU1805, Gh434, TMB2038, BNL2681, NAU2877, HAU1311
GO:0009628	Response to abiotic stimulus	12	NAU1063, MUSS281, NAU2257, NAU3008, NAU3234, NAU980, HAU3249, NAU5064, HAU2624, MUCS530, NAU3811, MON_CGR6766	7	HAU1794, MUCS347, HAU0720, HAU3342, NAU1366, HAU2026, HAU1311
GO:0010033	Response to organic substance	9	NAU3409, NAU3493, HAU1756, NAU1148, NAU3811, NAU2877, NAU5192, NAU3234, MUCS530	6	NAU3493, HAU1805, Gh434, TMB2038, BNL2681, NAU2877
GO:0009069	Serine family amino acid metabolic process	9	MUSS281, NAU2257, NAU3008, NAU980, MUSS92, MUSS404, NAU3748, NAU5354, NAU967	6	BNL2681, HAU2044, HAU0684, BNL137, NAU3748, HAU3342
GO:0051716	Cellular response to stimulus	8	DPL0715, MUSS281, NAU2257, NAU3008, NAU980, NAU3748, MUSB850, MUCS530	12	HAU1805, Gh434, TMB2038, NAU3748, HAU3303, MON_DPL0582, HAU1311, MUSB850, NAU2361, NAU2758, NAU3091, NAU3895
GO:1901700	Response to oxygen-containing compound	7	NAU3409, NAU3493, HAU1756, MUSS281, NAU2257, NAU3008, NAU980	6	NAU3493, HAU1805, Gh434, TMB2038, BNL2681, HAU1311
GO:0006970	Response to osmotic stress	7	MUSS281, NAU2257, NAU3008, NAU3234, NAU980, HAU3249, NAU5064	1	HAU3342
GO:0009651	Response to salt stress	7	MUSS281, NAU2257, NAU3008, NAU3234, NAU980, HAU3249, NAU5064	1	HAU3342
GO:0033554	Cellular response to stress	6	DPL0715, MUSS281, NAU2257, NAU3008, NAU980, MUSB850	7	HAU1805, HAU3303, Gh434, TMB2038, MON_DPL0582, HAU1311, MUSB850
GO:0070887	Cellular response to chemical stimulus	5	MUSS281, NAU2257, NAU3008, NAU980, MUCS530	4	HAU1805, Gh434, TMB2038, HAU1311
GO:0010035	Response to inorganic substance	5	MUSS281, NAU2257, NAU3008, NAU980, HAU1756	1	HAU1311
GO:0046686	Response to cadmium ion	5	HAU1756, MUSS281, NAU2257, NAU3008, NAU980	0	
GO:0010038	Response to metal ion	5	HAU1756, MUSS281, NAU2257, NAU3008, NAU980	0	
GO:0009719	Response to endogenous stimulus	4	NAU3493, HAU1756, NAU1148, NAU3811	5	NAU3493, HAU1805, Gh434, TMB2038, BNL2681
GO:1901701	Cellular response to oxygen-containing compound	4	MUSS281, NAU2257, NAU3008, NAU980	4	HAU1805, Gh434, TMB2038, HAU1311
GO:0009725	Response to hormone stimulus	4	NAU3493, HAU1756, NAU1148, NAU3811	4	NAU3493, HAU1805, Gh434, TMB2038
GO:0042542	Response to hydrogen peroxide	4	MUSS281, NAU2257, NAU3008, NAU980	3	HAU1805, Gh434, TMB2038
GO:0014070	Response to organic cyclic compound	4	NAU3493, NAU5192, MUCS530, HAU1756	3	HAU1805, Gh434, TMB2038
GO:0009416	Response to light stimulus	3	HAU2624, MUCS530, NAU3811	4	HAU0720, HAU3342, NAU1366, HAU2026
GO:0009314	Response to radiation	3	HAU2624, MUCS530, NAU3811	4	HAU0720, HAU3342, NAU1366, HAU2026
GO:0009611	Response to wounding	3	NAU2661, NAU3409, NAU3811	0	
GO:0009607	Response to biotic stimulus	2	NAU2877, NAU2809	6	BNL2681, NAU2877, HAU1805, Gh434, TMB2038, HAU3303
GO:0051707	Response to other organism	2	NAU2877, NAU2809	6	BNL2681, NAU2877, HAU1805, Gh434, TMB2038, HAU3303
GO:0009617	Response to bacterium	2	NAU2877, NAU2809	5	HAU1805, Gh434, TMB2038, NAU3653, NAU3373
GO:0033993	Response to lipid	2	NAU3493, HAU1756	4	NAU3493, HAU1805, Gh434, TMB2038
GO:0009266	Response to temperature stimulus	2	NAU1063, MON_CGR6766	3	HAU1794, MUCS347, HAU3342
GO:0009408	Response to heat	2	NAU1063, MON_CGR6766	2	HAU1794, MUCS347
GO:0009639	Response to red or far red light	2	HAU2624, MUCS530	2	HAU0720, NAU1366
GO:1901698	Response to nitrogen compound	2	HAU1756, MUCS530	1	BNL2681
GO:0071310	Cellular response to organic substance	1	MUCS530	3	HAU1805, Gh434, TMB2038
GO:0042594	Response to starvation	1	MUSB850	3	MON_DPL0582, HAU1311, MUSB850
GO:0009414	Response to water deprivation	0		1	HAU1311
GO:0009605	Response to external stimulus	1	MUSB850	4	BNL2681, MON_DPL0582, HAU1311, MUSB850
GO:0043549	Regulation of kinase activity	1	NAU3621	3	HAU1805, Gh434, TMB2038
GO:0009991	Response to extracellular stimulus	1	MUSB850	3	MON_DPL0582, HAU1311, MUSB850
GO:0006974	Response to DNA damage stimulus	1	DPL0715	0	
GO:0016458	Gene silencing	4	MUSB850, Gh074, DPL0715, MUCS530	2	MUSB850, HAU1592
GO:0006342	Chromatin silencing	2	MUSB850, DPL0715	1	MUSB850
GO:0007154	Cell communication	2	NAU3748, MUSB850	11	HAU1805, Gh434, TMB2038, NAU3748, MON_DPL0582, HAU1311, MUSB850, NAU2361, NAU2758, NAU3091, NAU3895
GO:0031047	Gene silencing by RNA	2	MUCS530, DPL0715	0	
GO:0007165	Signal transduction	1	NAU3748	8	HAU1805, Gh434, TMB2038, NAU3748, NAU2361, NAU2758, NAU3091, NAU3895
GO:0006952	Defense response	1	NAU2809	4	HAU1805, Gh434, TMB2038, HAU3303
GO:0010941	Regulation of cell death		0	4	HAU1805, HAU3303, Gh434, TMB2038
GO:0019932	Second-messenger-mediated signaling		0	3	NAU2361, NAU2758, NAU3091

## Discussion

The approach in this study was to develop a genetic and physical framework for the genomic regions of Upland cotton homoeologous Chr 11 and Chr 21 that contain important nematode and fungal disease resistance associations with molecular markers such as SSRs. While various QTL and other genetic mapping approaches have revealed the importance of this pair of cotton chromosomes in defense to biotic stresses, there has hitherto been little physical structure development and use of sequence annotation to advance our understanding of its genetic organization. The current and previous marker work provided numerous mapped marker sequences for these two chromosomes, some of which are important for use in cotton breeding programs. Furthermore, this resource allowed us to identify existing BAC clones in the *G. hirsutum* Acala Maxxa BAC library that are from Chr 11 and Chr 21 based on genetic mapping with SSR markers derived from the BAC-end sequences. Targeted full clone sequence of these mapped BAC clones provided a second resource of genomic DNA sequence to investigate defense response motif content of this cotton genome region. The Maxxa BAC clone and marker sequence data were also compared to the whole genome sequence assemblies of the *G. raimondii* D_5_ and *G. arboreum* A_2_ ancestral diploid genomes (Paterson et al., 2012; Li et al., 2014), and two *G. hirsutum* TM-1 AD_1_ whole genome assemblies which are now publicly available (Li et al., 2015; Zhang et al., 2015).

Of particular interest is the very high defense response element content of sequences from both the SSR markers and the BAC clones on both Chr 11 and Chr 21. This result is in line with the currently recognized importance of this pair of cotton chromosomes in resistance to a wide range of parasitic nematodes and disease-causing pathogens of cotton revealed through genetic mapping of resistance trait determinants. The gene ontology annotations clearly demonstrate the richness of this region in the evolution of defense genes. Typically resistance loci evolve by tandem duplication followed by mutation and divergence of functional specificity, for example nematode resistance in soybean (Cook et al., 2012), often in response to or as a hedge against similar mutation and evolutionary changes in virulence factors in the nematode or pathogen. The large number of NBS-LRR type motifs with tandem repeats, for example as summarized for one of the two BAC clones in Figure 2 and sequence duplication of the BAC clones on Chr 21 (Figure 2), exemplifies this evolutionary hot-spot of defense gene-rich arrangement.

Comparison of DNA sequence between Chr 11 and Chr 21 for certain BAC clones also indicates the high homology between the sequences of the homoeologous chromosome pair. Thus, herein we not only report apparent large-scale duplication events within an Upland cotton chromosome, but also considerable duplication and an evolving separation of sequence homology between a pair of homoeologous chromosomes. This provides cotton with an enormous reservoir of defense response genes, some of which may be defeated related to prior pathogen forms, while others provide a resource for defense against future pathogen forms.

More TEs were identified on Chr 11 (At subgenome) than on Chr 21 (Dt subgenome) according to both *G. hirsutum* Unigene (A/D: 44/9) and predicted gene databases (A/D: 56/16) (Table 1), which might account for the physical difference in size of the A-subgenome in reference to the D-subgenome. Li et al. (2014) reported that there were a total of 4098 TEs on Chr 7 (equivalent to Chr 11 in *G. hirsutum*) in the diploid *G. arboreum* A genome and only 1542 TEs on Chr 7 (equivalent to Chr 21 in *G. hirsutum*) in the diploid *G*. *raimondii* D_5_ genome even though there were similar numbers of loci identified on Chr 7 in both diploid genomes. At least 64.8% TEs were identified in the TM-1 genome by Zhang et al. (2015) and 66% TEs by Li et al. (2015). More TEs in the A sub-genome (at least 843.5 Mb, genome size 1477 Mb) than in the D sub-genome (at least 433 Mb, genome size 831 Mb) were determined in the TM-1 genome (Zhang et al., 2015). TEs are known to play a dominant role contributing to angiosperm evolution and diversity (Oliver et al., 2013). In cotton, allotetraploid *G. hirsutum* was derived from reuniting of diploid A and D genomes about 1–2 million years ago (mya) through independent and differential accumulation of TEs 5 mya (Hu et al., 2010; Li et al., 2014). We found that resistance genes in BACs were always surrounded with retrotransposable elements (Table 3). Retrotransposons based on “cut and paste” mode are more abundant in cotton, including Ty1-copia and Ty3-gypsy elements (Hawkins et al., 2006; Hu et al., 2010). More than 50% retrotransposon frequencies were reported in the TM-1 genome (Li et al., 2015; Zhang et al., 2015). TEs involved in abiotic and biotic stress responses have gained more attention recently (Grandbastien, 1998; Grandbastien et al., 2005; Cowley and Oakey, 2013; McDowell and Meyers, 2013; Oliver et al., 2013; Tsuchiya and Eulgem, 2013; Wheeler, 2013). More TEs on the At subgenome might suggest more adaptation to biotic stress response on Chr11 than on Chr 21. In addition, we found 120 DNA-RNA polymerase family protein genes contributing to regulation of transcription on Chr 11 BACs with the *G. hirsutum* Unigene database but none of these on Chr 21. It is not clear to what extent DNA-RNA polymerase family proteins function in stress response but these results suggest divergent evolution between the A and D genomes.

Comparison of *G. hirsutum* AD_1_ whole genome with A_2_ and D_5_ were thoroughly conducted by Li et al. (2015) and Zhang et al. (2015) and with other genomes (*A. thaliana, T. cacao, Glycine max, and V. vinifera*) (Li et al., 2015). However, the 20 Maxxa BACs could not be fully mapped to the TM-1 genome, indicating that differences occur between the two tetraploid *G. hirsutum* AD_1_ cotton varieties. Abundant transposable elements might cause the difference between the two *G. hirsutum* cotton varieties. In addition, homeologous exchanges were also observed between At subgenome Chr 11 and Dt subgenome Chr 21 (Tables S14, 15). For example, Maxxa BAC 32H19 linked to MUSB0823 on Chr 21 (Figure 1, Yu et al., 2012) was mapped to both Chr11 and Chr 21on TM-1 genome (Tables S14, S15).

Comparisons between Maxxa BACs from the tetraploid AD_1_ cotton and the A_2_ and D_5_ ancestral genomes were made to better understand the evolution of the AD genome, particularly in regard to relationships that may shed light on resistance evolution. Comparison of sequence alignments showed less similarity between tetraploid AD Chr 11 and D_5_ genome than between AD Chr 21 and D_5_ genome, further supporting independent evolution of the A and D genomes. Likewise, sequence alignments showed less similarity between tetraploid AD Chr 21 and A_2_ genome than between AD Chr 11 and A_2_ genome. The divergence of the A and D genomes is also reflected in the origins of resistance traits. For example, in a previous study *G. hirsutum* (AD_1_) and *G. barbadense* (AD_2_) were found to share the same SSR marker MUCS088 alleles as *G. arboreum* (A_2_), suggesting nematode resistance introduction was from the diploid cotton (A_2_) genome (Roberts and Ulloa, 2010).

The comparison of aligned sequences with four other sequenced plant taxa indicated a conservation of genic sequence among these plants. The highest similarities of cotton BAC sequence to the other plant taxa indicated the closest relationship with *T. cacao*. Both *G. raimondii* and *G. arboreum* genomes showed close collinear relationships with *T. cacao* and both of them might share a common ancestor having diverged from *T. cacao* 18–58 mya (Paterson et al., 2012; Wang et al., 2012b; Li et al., 2014).

Genome-wide association studies (GWAS) have been utilized successfully to identify genetic variation in plants (Brachi et al., 2011), and the availability of diploid and tetraploid whole genome sequences makes possible GWAS for identifying genetic variation in cotton. A whole genome marker map in cotton was constructed by Wang et al. (2013) based on the *G. raimondii* D_5_ genome (Paterson et al., 2012). Wei et al. (2013) conducted systematic analysis and comparison of nucleotide-binding site disease resistance genes in the *G. raimondii* D_5_ genome (Wang et al., 2012b) and genome-wide analysis of the gene families of resistance gene analogs and their response to Verticillium wilt was made in both the *G. raimondii* D_5_ (Chen et al., 2015) and *G. arboreum* A_2_ genomes (Li et al., 2014). A comprehensive meta QTL analysis was made for fiber quality, yield, drought tolerance and disease resistance with different cotton populations (Said et al., 2013). GWAS in the tetraploid (AD) TM-1 cotton revealed positively selected genes for fiber improvement in the A genome and for stress tolerance in the D genome (Zhang et al., 2015). GWAS in the allotetraploid cotton to identify resistance-rich regions will provide more insights about the evolution of the homoeologous chromosomes 11 and 21 and benefit disease management.

In conclusion, the sequence information and physical mapping of BAC clones provide an additional genomic resource of these resistance gene-rich regions of the Upland cotton genome on Chr 11 and Chr 21. BAC clone sequences are deposited in GenBank (NCBI: http://www.ncbi.nlm.nih.gov). Continuing genetic and physical framework alignment of sequence information in cotton will help to expedite the discovery of R and PR genes and the assembly of a whole Upland cotton tetraploid genome, eventually supporting breeding for disease resistance in cotton production.

### Conflict of interest statement

The authors declare that the research was conducted in the absence of any commercial or financial relationships that could be construed as a potential conflict of interest.
